# Editorial for the Special Issue “Feature Papers in Chemistry and Physics of Biological Gels”

**DOI:** 10.3390/gels11060417

**Published:** 2025-05-30

**Authors:** Esmaiel Jabbari

**Affiliations:** Biomimetic Materials and Tissue Engineering Laboratory, Department of Chemical Engineering, University of South Carolina, Columbia, SC 29208, USA; jabbari@engr.sc.edu

We are excited to share with Gels’ community the Special Issue on the chemistry and physics of biological gels. The contributions in this Special Issue showcase gels with novel chemistry and physical properties for drug delivery, regenerative medicine, and wound healing. The unique physical, mechanical, and biological properties of these natural gels stem from their complex structures at different length scales, spanning from the nanoscale, with van der Waals, acid-base, and electrostatic interactions, to the mesoscale, with secondary and tertiary interactions, followed by the microscale, with fibrillar and fibrous structures.

A novel application of biological gels is in 3D printing for the controlled deposition of cells and the extracellular matrix (ECM) in creating complex biological tissues. Ivan Donati and collaborators [1] studied the addition of silk fibroin (SF), which is a natural fibrous protein extracted from cocoons of Bombyx mori, to alginate methacrylate (ALMA) and gelatin on rheological properties, printing stability, and encapsulated cell viability in the produced hydrogels. Hydrogel precursors were successfully crosslinked via laser-light-assisted gelation via the methacrylate groups of ALMA. They reported that SF-enriched hydrogels exhibited greater elasticity and excellent fibroblast cell viability as compared those without SF. This novel bioink could potentially produce models for long-term cell culture for bone and cartilage tissue regeneration.

Collagen is used extensively as a matrix for tissue reconstruction in medicine. Stem cells seeded in preformed collagen scaffolds or encapsulated in collagen gels have shown great promise in cranial and maxillofacial bone repair and dental applications. Thibaud Coradin and collaborators [2] studied the effect of collagen fibrillogenesis on matrix stability, cell viability, and mineralization of encapsulated human dental pulp stem cells (hDPSCs). Collagen fibrillogenesis was controlled by varying acetic acid and buffer concentrations before and after plastic compression, This work demonstrates that the suitability of a collagen matrix for cell delivery in regenerative medicine is dependent on aging conditions, like the acetic acid concentration and cell culture medium concentration. The cell type and density also affect the extent of extracellular matrix deposition via the seeded stem cells.

Carbohydrate-based amphiphiles self-assemble to form supramolecular gels with desirable biodegradability and biocompatibility. Pablo Hector Di Chenna, Maria Laura Uhrin, and collaborators [3] studied self-assembly, gelation, and wheat lectin binding of amphiphilic compounds having β-S-N-acetylglucosamine residues linked to 2,3-diacyl-N,N′-dipropargylated-l-tartaric diamide. A click reaction was used to synthesize the two amphiphiles, which differed in the length of the fatty acid attached to positions 2 and 3 of the tartaric acid scaffolds. The presence of a fibrillar morphology confirmed that these carbohydrate-based amphiphiles form physical gels. The authors reported that the amphiphiles with short non-polar chains had optimal solubility for self-assembly in water in a chiral arrangement to form a stable hydrogel with a minimum hydrogelator concentration. This contribution advances our knowledge of structural parameters that impact the self-assembly of carbohydrate-based amphiphiles for important applications in immunotherapy and gluten sensitivity management.

Hydrogels based on natural polymers of hyaluronic acid and chitosan are widely used in wound dressings due to their complementary biocompatibility and degradability. Honey contains high glycine, methionine, arginine, and proline concentrations, which are indispensable amino acids for facilitating fibroblast deposition and collagen synthesis in wound healing. Honey has been used in traditional medicine for its antibacterial properties. Emin Salva and collaborators [4] studied the effect of adding honey to chitosan–hyaluronic acid gels on crosslinking, water content, cell adhesion, and proliferation of fibroblasts. The authors report that water content and porosity of the gels decreased with the addition of honey, and fibroblast cell adhesion to the gels improved with the addition of honey. The authors demonstrate that honey-loaded chitosan–hyaluronic acid solutions forms gels in the absence of covalent crosslinkers and the formed gels improve fibroblast cell adhesion which could be beneficial to wound healing.

Enguerran Devernois and Thibaud Coradin [5] reviewed the synthesis and biological properties of type I collagen–chitosan-mixed hydrogels. Collagen as a scaffold for tissue regeneration provides excellent biocompatibility and biochemical cues for cell adhesion and proliferation, but its use is limited by its inadequate mechanical stability. Conversely, chitosan has flexible mechanical properties that can be tuned to a specific application, but it is not the optimal substrate for cell adhesion and growth. In this review, the authors highlight the benefits of collagen–chitosan-mixed hydrogels in terms of rheological properties, physical, chemical, or enzymatic crosslinking, gel structure, mechanical and surface properties, and cell interaction. The authors indicate that little is known about the interactions of type I collagen with chitosan which should be investigated in future works.
